# Bacterial diversity in the freshwater sponges of Sundarban and their potential role in biomonitoring toxic element pollution

**DOI:** 10.1128/spectrum.02149-25

**Published:** 2025-08-27

**Authors:** Dhruba Bhattacharya, Namrata Jiya, Sangita Mondal, Agnita Acharya, Abhijit Chatterjee, Utpal Bakshi, Avinash Sharma, Abhrajyoti Ghosh

**Affiliations:** 1 Department of Biological Sciences, Bose Institute 30141, Kolkata, India; 2 BRIC-National Centre for Cell Science, Pune, India; 3 Department of Chemical Sciences, Bose Institute 30141, Kolkata, India; 4 Instistitute of Health Sciences, Presidency University 421906, Kolkata, India; University of Minnesota Twin Cities, St. Paul, Minnesota, USA

**Keywords:** freshwater sponges, microbiome, Sundarban, potentially toxic elements (PTE), metataxonomics

## Abstract

Sponges serve as the natural cleaner in diverse aquatic ecosystems, harboring diverse microbial communities and forming a highly specialized holobiont. The present study provides the first insights into the bacterial communities associated with freshwater sponges of Sundarban, highlighting their distinct microbial community composition compared to the surrounding water using 16S rRNA gene-based metataxonomic analyses. The analysis encompassing six sponge species collected from Sagar Island and Ghoramara revealed distinct variations in microbial abundance and diversity compared to their ambient water, suggesting selective bacterial associations with the sponges. The sponge species displayed a varied abundance of bacterial taxa across different species and locations. Furthermore, significantly greater concentrations of potentially toxic elements (PTEs) were observed in sponges when compared with corresponding water samples, thereby suggesting the bioaccumulation capability of these sponges. Functional profiling of bacterial communities in sponge and water samples revealed enrichment of various metabolic pathways linked to metal ion transport and resistance, as well as antimicrobial resistance in the sponges, suggesting an adaptive response to environmental stressors. The abundance of diverse metal ion regulators and transporters underscores the ecologically significant function of sponge-associated bacterial communities in metal ion resistance, placing the sponges as promising bioindicators for monitoring polluted aquatic ecosystems.

**IMPORTANCE:**

Freshwater sponges play an essential role as natural biofilters in aquatic ecosystems, effectively purifying water. These sponges harbor unique microbial communities, forming a holobiont that is key to their ecological function. This study offers new insights into the bacterial communities associated with freshwater sponges from the Sundarbans, a previously underexplored region. Using 16S rRNA gene-based metataxonomic analyses, we compared the bacterial diversity of six sponge species from Sagar Island and Ghoramara to that of surrounding water. Our results reveal distinct bacterial associations within sponges, different from the ambient water’s microbial composition. Notably, sponge concentrations of potentially toxic elements (PTEs) were much higher than in the surrounding water, highlighting their bioaccumulation capacity. Functional profiling of sponge-associated bacteria revealed genes related to metal ion transport and antimicrobial resistance, suggesting adaptive responses to environmental stress. This research enhances our understanding of sponge microbiomes and their potential for bioremediation, particularly for removing heavy metals from polluted water.

## INTRODUCTION

Sponges (phylum Porifera) are sessile filter-feeding organisms that rank among the oldest living multicellular animals inhabiting various aquatic environments worldwide. Sponges actively pump and filter substantial water through their specialized aquiferous system (1). The remarkable filtering capacity positions sponges as critical players in aquatic ecosystems, contributing to nutrient cycling and linking the benthic and pelagic zones. By recycling vital elements such as carbon, nitrogen, and silicon, sponges play crucial ecological, evolutionary, and microbiological roles, highlighting their prominence in freshwater and marine environments (2, 3). Apart from offering habitat and protection to various microalgae, microinvertebrates, and microeukaryotes, sponges also host phylogenetically diverse, species-specific prokaryotic symbiotic assemblages (4–7). Some high microbial abundance marine sponges (HMA) can contain up to 40% microorganisms by weight (8). Sponge symbionts are involved in a variety of metabolic processes, including nitrogen fixation, nitrification, photosynthesis, and sulfate reduction (9–12), likely contributing to the nutritional needs of host sponges (13). Furthermore, some microbial symbionts produce an array of secondary metabolites that may play a role in the chemical defense mechanisms of their host sponges (14, 15). Consequently, host sponges and their associated microbiota are often referred to as a “holobiont,” representing a unique biological entity with a shared genetic makeup (16).

Although several studies have explored the microbial associations of marine sponges, which comprise over 95% of known marine sponge species, reports on freshwater sponges remain limited. While freshwater sponges perform similar ecological functions, only sporadic studies have examined their microbiomes, impeding investigations into sponge-microbe specificity and metabolic relationships influenced by environmental factors. Notably, research has focused on the endemic sponge *Lubomirskia baicalensis* from Lake Baikal, including its diseased individuals (17–24). In contrast, data on the microbial diversity of other freshwater sponge species, such as *Corvospongilla lapidosa* (25), *Eunapius carteri* (25), *Tubella variabilis* (26), *E. muelleri* (27), *Spongilla lacustris* (28–30), *Ephydatia fluviatilis* (1), and *Metania reticulata* (31), remain sparse. Out of these, only a few studies reported the next-generation sequencing analyses (1, 17, 19, 20, 22, 25, 27, 32) resulting in limited high-resolution taxonomic data. Additionally, no studies have compared the freshwater sponge microbiomes to ambient samples like water samples from sponge habitats, which is a crucial factor to determine whether these microbiomes reflect distinct planktonic or benthic communities rather than specific host-microbe associations. The limited data available leave key hypotheses regarding sponge-microbe specificity, metabolic networks, and environmental factors shaping microbiome structure largely unexamined in freshwater sponges. Investigating the microbiomes of other freshwater sponge species, alongside ambient water samples from various ecosystems, could provide valuable insights into conserved sponge-microbe relationships. Moreover, it may shed light into the role of such a complex “holobiome” in environmental management. Freshwater sponge research in India is steadily gaining attention, revealing the biotechnological and ecological contributions of their associated microbial communities. For instance, the microbiota of *Eunapius carteri* and *Corvospongilla lapidosa* were explored using next-generation sequencing, revealing a diverse microbial composition covering 14 phyla and up to 2,900 OTUs, which, in some cases, surpass the microbial diversity typically observed in marine sponges (25). Similarly, sponges from the Little Rann of Kutch, including *Spongilla* and *Ciocalypta*, were shown to host a diverse range of microbial taxa, with 376 OTUs from 41 phyla, and evidence of anammox-planctomycetes, including novel species related to *Candidatus Brocadia* (33). These studies collectively underline the rich and distinct microbial assemblages in freshwater sponges studied so far from India, which are significantly different from marine counterparts and assuring biotechnological potential.

The Sundarban Delta, located in the western part of the Ganges-Brahmaputra Delta, is the world’s largest contiguous tidal mangrove ecosystem, home to unique halophytic flora and endangered fauna (34). This ecosystem, positioned between pristine southern mangroves and urbanized northern areas, faces substantial industrial effluents and land conversions, resulting in the accumulation of potential toxic elements (PTE) in estuarine water, sediments, and aquatic organisms. The pollution caused by PTEs is one of the major threats to the mangrove ecosystem of Sundarban. Several reports (35, 36) indicated the moderate to heavy PTE contamination in the sediments and water of this vital ecosystem (37–39). PTEs like cadmium (Cd), copper (Cu), iron (Fe), and zinc (Zn) chiefly originate from the anthropogenic activities, including industrial processes, ferry operations, and aquaculture practices posing significant risk of severe ecotoxicological effects (38). High bio-accumulation capacity and toxicity of these PTEs not only raise serious concern about biodiversity loss but also can cascade the changes in ecosystem stability.

Sponges filter vast amounts of water and can accumulate trace metals; therefore, they are commonly regarded as sentinels for toxic elements (40, 41). This characteristic makes them practical bio-monitoring tools in aquatic systems, where they can indicate significant trace metal concentrations near contaminant sources (42–46). Their role as native fauna also helps reduce the risk of introducing invasive species. Recently, sponges have become valuable model species for assessing various organic pollutants, including xenobiotic compounds and PTEs, and are acknowledged as biomarkers for several pollutants (42, 47, 48). However, the knowledge about their capacity to detect and evaluate many xenobiotic pollutants remains limited.

This study sought to explore the bacterial diversity in six sponges collected from the shallow waters of Sagar Island and Ghoramara in the Sundarbans, along with ambient water samples. It also measured several potentially toxic elements (PTEs) in water and sponge samples. This is the first report on the microbial diversity of sponges in the Sundarbans. Functional prediction analysis was also performed on the bacterial communities in sponges and water to investigate processes related to metal ion transport, resistance mechanisms, and antimicrobial resistance.

Studying sponges from Sagar Island and Ghoramara is crucial as these sponges are exposed to distinctive environmental conditions impacted by both natural and anthropological activities, including rapid habitat changes and pollution. Analyzing these sponges and their associated microbiomes can reveal important aspects of microbial diversity, bioaccumulation of potentially toxic elements, and ecosystem health, contributing to a comprehensive understanding of biodiversity and environmental resilience in the Sundarbans. From the health perspective, this research can indicate how the pollution affects the environment, animals and humans, especially in areas with high levels of PTEs.

## MATERIALS AND METHODS

### Sample collection

**Fig 1:**
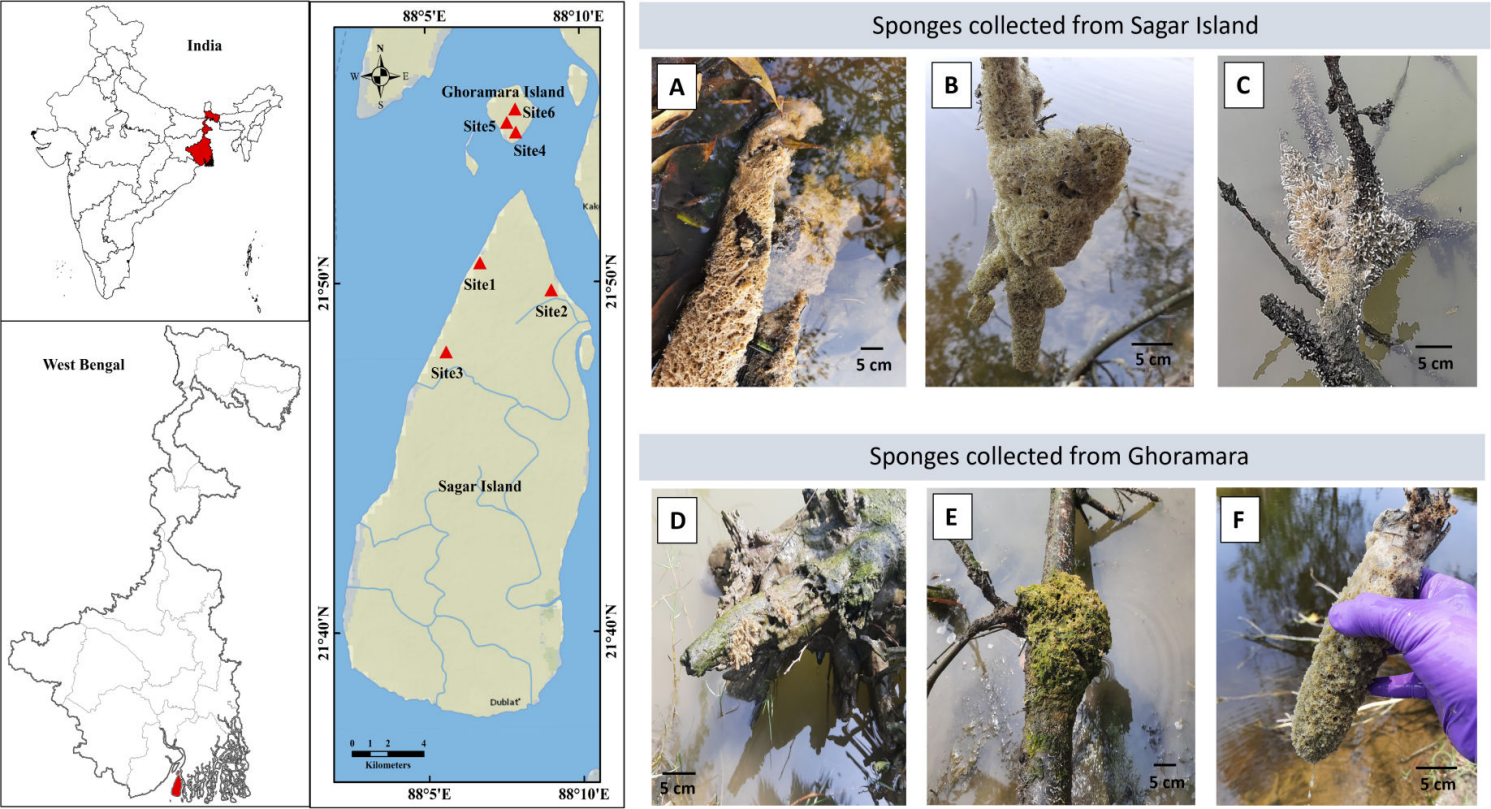
Geographical map and freshwater sponge specimens collected from the Sundarban. Left panel: Map showing the two islands in the Sundarban delta with sampling sites labeled in collection order. Right panel (top): Photographs of sponges *Spongilla alba* (**A**), *Eunapius carteri* (**B**), and *Ephydatia fluviatilis* (**C**), collected from sampling sites 1, 2, and 3 on Sagar Island, respectively. Right panel (bottom): Photographs of sponges *Radiospongilla cerebellata* (**D**), *Ephydatia meyeni* (**E**), and *Spongilla alba* (**F**), collected from sampling sites 4, 5, and 6 on Ghoramara Island, respectively.

A total of six sponge samples were collected from the Sundarban Delta, with three from the shallow water bodies of Sagar Island (21°50′34.8″N 88°06′44.6″E, 21°49′48.0″N 88°09′02.5″E, 21°48′01.7″N 88°05′39.3″E) and three from Ghoramara Island (21°54′18.6″N 88°07'54.9″E, 21°54′34.2″N 88°07′38.5″E, 21°54′58.3″N 88°07′56.0″E) (Fig. 1). The sampling was conducted during March–April 2022. A sterile spatula, scalpel, and gloves were used during collection, and the sponge samples were kept in sterile zip-lock bags. Alongside the sponge samples, water samples from the same water bodies were also collected in sterile glass bottles. Three biological replicates were collected for each sponge and water sample. At each site, 1 L of water was collected in triplicate within 25 cm radius of the freshwater sponges. Soon after collection, the physicochemical parameters of the samples, such as pH, conductivity (mS/cm), salinity (ppt), total dissolved solids (TDS) (mg/L), resistivity (Ω⋅m), and dissolved oxygen concentration (mg/L), were measured with a handheld Eutech PCD 650 multiparameter meter (Thermo Fisher Scientific, United States). The sponge samples were stored in a −80°C freezer until further processing.

### Identification of the sponges

The sponge samples were primarily identified based on spicule morphology using a confocal microscope (Leica, Germany). For confocal microscopic observations, permanent slides of both body and gemmule spicules were prepared for each sponge sample by digesting small sponge fragments in concentrated nitric acid (HNO₃), followed by dehydration using ethanol, and mounting in Distyrene Plasticizer Xylene (DPX liquid mountant; HiMedia). The sponge samples were identified based on detailed analysis of spicule morphology, following comparative evaluation with descriptions reported in existing literature (49–52). Additionally, molecular identification of the sponges was performed through sequencing of the D3 domain of 28S rDNA, following the protocol described by Lopp et al. (53). The Sanger sequencing reads were aligned using BioEdit software (54). The resulting sequences were submitted to the NCBI GenBank database under accession numbers PV413050 to PV413055. For phylogenetic analysis, multiple sequence alignment was performed using ClustalW (55), and a Neighbor-Joining (NJ) tree was constructed in MEGA11 (56) to infer the evolutionary relationships among the sponge species.

### Measurement of potential toxic elements concentrations

Concentrations of several potentially toxic elements (PTEs), including Arsenic (As), Zinc (Zn), Iron (Fe), Copper (Cu), Lead (Pb), Cadmium (Cd), and Chromium (Cr), were measured in both sponge and water samples. For water samples, digestion was done by adding 0.5 mL concentrated HNO_3_ into 10 mL of each water sample and heating at 105°C for around 2 h 30 min until a colorless solution was obtained. The resultant acid-digested solution was cooled and diluted to 10 mL with ultrapure water (18.2 mS/cm). Similarly, each sponge sample, weighing 0.5 g, was first lyophilized to eliminate water and then digested using 5 mL of concentrated HNO_3_. Acid-digested samples were diluted by adding ultrapure water, making the final volume of 25 mL. Afterward, the acid-digested water and sponge samples were filtered through Axiva 0.2 µm PTFE filter paper (Axiva Sichem, India) and stored at 4°C in 15 mL polypropylene tubes for further analysis.

The concentrations of PTEs were estimated using an Inductively-coupled plasma optical emission spectrometer (ICP-OES) (iCAP 7400 ICP-OES analyzer; Thermo Fisher Scientific, United States) following the method as suggested by American Public Health Association (57). All the plasticware and glasswares used in this analysis were washed with 10% (vol/vol) HNO_3_ solution followed by ultrapure Milli-Q water and air dried. Calibration standard solutions were prepared by using ICP Multi-elemental Standard Solution IV and IX (Merck, Germany). The quality of the analytical process in ICP-OES was compared with the analysis of certified standard reference material from the National Institute of Standards and Technology (NIST), United States. The data on the concentrations of PTEs in both water and sponge samples were analyzed in R version 4.4.1. The PTE concentration values were log-transformed to visualize the concentration ranges better. Additionally, the Wilcoxon test was performed to compare statistically between sample types.

### Sample preparation and DNA extraction

Sponge samples were cut into smaller fragments, and the surface tissue layers were aseptically scraped off using a sterile scalpel to remove detritus and planktonic microorganisms. Approximately 1 g of internal tissue was collected from each sample for DNA extraction. Water samples were filtered using cellulose-acetate membrane filter discs with a 0.2 µm pore size (Sartorius, Germany) before DNA extraction, and the bacterial community DNA was extracted directly from the filter discs. Following the manufacturer’s protocol, total microbial genomic DNA from the sponge and water samples was extracted using the NucleoSpin Soil kit (Macherey-Nagel, Germany). The quality and concentration of extracted DNA from each sample were checked using a NanoDrop One Microvolume UV-Vis spectrophotometer (Thermo Fisher Scientific, United States).

### Targeted 16S rRNA gene sequencing

The extracted microbial community DNA was used as a template for the amplification of the hypervariable V4 region of 16S rRNA using the primers 515F (5′-GTGYCAGCMGCCGCGGTAA-3′) and 806R (5′-GGACTACNVGGGTWTCTAAT-3′) (58). The PCR thermocycling protocol included an initial denaturation at 95°C for 5 min, followed by 30 cycles consisting of denaturation at 95°C for 30 s, annealing at 55°C for 30 s, and extension at 72°C for 30 s, with a final extension step at 72°C for 5 min. The amplicons were purified with AMPure XP beads (Beckman Coulter, United States). The concentrations of purified amplicons were measured with a Qubit 3.0 Fluorometer (Thermo Fisher Scientific, United States). Equal amounts of each amplified sample were normalized and then pooled in equimolar proportions. The paired-end sequencing was performed on the Illumina MiSeq platform using 2 × 250 bp reads per end with V2 chemistry. The raw sequence reads were submitted to NCBI Sequence Read Archive (SRA) with BioProject accession number PRJNA1101956.

### Processing of 16S rRNA gene sequence data

The raw paired-end reads were processed using QIIME2 version 2024.5 (59). The demultiplexed paired-end reads were imported into QIIME2. The quality of the reads was summarized, and denoising was performed employing the DADA2 algorithm (60) with trimming parameters set at 19 bp (forward) and 20 bp (reverse) in order to remove the primer sequences. Denoised sequences were used to create a feature table and representative sequences. The SILVA 138.1 database (61) was used for taxonomic classification by retrieving the SSURef NR99 sequences, followed by reverse-transcribing RNA to DNA, culling sequences, filtering by taxon-specific lengths, and finally dereplicating sequences. Primers (515F/806R) were applied to extract the relevant reads. Subsequently, the classifier was trained with the Naive Bayes algorithm. A reference phylogenetic tree was constructed using SEPP insertion in QIIME2 (62).

### Bioinformatics and statistical analysis

The QIIME2 artifacts, including OTU tables, taxonomy, and phylogenetic tree files, were imported and analyzed in R version 4.4.1 (2024-06-14 ucrt). The microbial diversity analysis was conducted using the MicrobiotaProcess R package (63). After making the MicrobiotaProcess object from QIIME2 data, it was filtered to remove unwanted taxa such as Archaea, mitochondria, and chloroplasts. Rarefaction was performed with the *mp_rrarefy* function to standardize sequencing depth, followed by calculating rarefaction curves using the *mp_cal_rarecurve* function. The alpha diversity metrics, including observed richness, Chao1, Shannon, and Pielou indices, were calculated using the *mp_cal_alpha* function. Phylogenetic diversity metrics viz. PD, NRI (Net Relatedness Index), and NTI (Nearest Taxon Index) were computed using *mp_cal_pd_metric* to explore phylogenetic relationships within microbial communities in water and sponge samples. The relative abundance of taxa was calculated using the *mp_cal_abundance* function. Abundant phyla present in water and sponge samples of Sagar and Ghoramara islands were estimated based on their relative abundance, and the result was plotted using the function *mp_plot_abundance*. For ordination analysis, the abundance data were normalized with the Hellinger transformation. The normalized data were then used for computing Bray-Curtis dissimilarity with the *mp_cal_dist* function. The Principal Coordinate Analysis (PCoA) was performed on the Bray-Curtis distance matrix using the *mp_cal_pcoa* function to investigate the pattern in the microbial diversity of sponge and water samples. The Bray-Curtis dissimilarity metric was used to calculate compositional differences between and within the sponge and water samples, and the group-level comparisons of sample types were statistically tested using the integrated group test function. Similarly, Permutational multivariate analysis of variance (PERMANOVA) was conducted using Bray-Curtis distances. Moreover, the Canonical Correspondence Analysis (CCA) was performed to evaluate the influence of physicochemical parameters and concentrations of PTEs on microbial community composition in water and sponge samples across different locations. The relationship between microbial diversity and these physicochemical factors was statistically evaluated through permutation-based ANOVA with 9999 permutations. The differential abundance analysis of microbial genera was carried out using the *mp_diff_analysis* function. The analysis employed the Kruskal-Wallis test followed by the Wilcoxon test to evaluate differences in the abundance of genera between sponge and water samples. Linear discriminant analysis (LDA) of effect size or LEfSe (64, 65) was used to identify and rank the genera that were best differentiated between the sample types. The significance threshold values were set at a false discovery rate (FDR) of 0.05 and a *P*-value of 0.05. A correlation analysis was performed using the microeco R package (66) to study the relationships between microbial community composition and environmental factors such as physicochemical parameters and PTE concentrations across the sponge and water samples.

### Prediction of functional genes

The functional gene abundance associated with metal ion transport and resistance (MITR) and antimicrobial resistance (AMR) was analyzed using Phylogenetic Investigation of Communities by Reconstruction of Unobserved States (PICRUSt2) (67). PICRUSt2 was installed as a QIIME2 plugin. The relative abundance profile of predicted Kyoto Encyclopedia of Genes and Genomes (KEGG) ortholog (KO) genes was annotated manually, and the KO (KEGG Orthology) identifiers with functions related to MITR and AMR were selected for further analysis. The functional differences in microbial communities associated with water and sponge samples were investigated with the ALDEx2 package (68). For each functional category (MITR and AMR), the ALDEx2 analysis was performed with the *aldex* function, which computed a *t*-test across the sample groups water and sponge. The log2 fold change of the MITR and AMR-related genes between sponge and water samples was visualized. All the plots were generated using the ggplot2 R package (69).

## RESULTS

### Morphological identification and habitat parameters of sponges

Primary identification of the collected sponge samples was carried out based on spicule morphology. This morphological analysis led to the identification of *Spongilla alba* (SP1), *Eunapius carteri* (SP2), and *Ephydatia fluviatilis* (SP3) from Sagar Island, and *Radiospongilla cerebellata* (SP4), *Ephydatia meyeni* (SP5), and *Spongilla alba* (SP6) from Ghoramara Island (see Fig. S1 at https://github.com/AG-Lab-BI/Freshwater_Sponges_Sundarban/blob/main/supplemental%20files.pdf). The alignment of 28S rDNA sequences of sponges revealed high sequence similarity across all samples, with a few variable regions (see Fig. S2 at https://github.com/AG-Lab-BI/Freshwater_Sponges_Sundarban/blob/main/supplemental%20files.pdf) that contributed to phylogenetic resolution in the NJ tree (see Fig. S3 at https://github.com/AG-Lab-BI/Freshwater_Sponges_Sundarban/blob/main/supplemental%20files.pdf). The tree revealed distinct clustering patterns among sponge taxa, with high bootstrap support values confirming the reliability of major clades (see Fig. S3 at https://github.com/AG-Lab-BI/Freshwater_Sponges_Sundarban/blob/main/supplemental%20files.pdf). The physicochemical parameters (see Table S1 at https://github.com/AG-Lab-BI/Freshwater_Sponges_Sundarban/blob/main/supplemental%20files.pdf) showed that the salinity of the samples ranges from 6.3 to 16.7 ppt, which clearly falls within the brackish water range (0.5–30 ppt), typical of estuarine environments. This suggests intrusions of saline water into these shallow water bodies. The pH range of 7.3–8.4 revealed that the samples are slightly alkaline. The pH and salinity values are slightly higher in Sagar Island samples than in the samples collected from Ghoramara. Lower TDS values, ranging from 5.4 to 6.8 mg/L, indicate relatively clean freshwater conditions in the Ghoramara samples. Conversely, the samples collected from Sagar Island displayed greater conductivity levels (10.4–13.9 mS/cm) and TDS values ranging from 10.1 to 12.4 mg/L, signifying the conversion toward further brackish conditions.

### PTE accumulation and concentrations

For all PTEs measured, sponge samples exhibited significantly higher concentrations than water samples (see Fig. S4 at https://github.com/AG-Lab-BI/Freshwater_Sponges_Sundarban/blob/main/supplemental%20files.pdf). The differences in As, Cr, Cu, Fe, and Zn concentrations were notable, with significant heavy metal sequestration patterns observed between the sponges and their ambient water samples (see supplemental Tables S1 and S2 at https://github.com/AG-Lab-BI/Freshwater_Sponges_Sundarban/blob/main/supplemental%20files.pdf).

### Microbial diversity and community richness

Sequencing depth and the richness of microbial communities in sponge and water samples were estimated with rarefaction curves showing the observed OTUs, Chao1, and Abundance-based Coverage Estimator (ACE) indices (see Fig. S5 at https://github.com/AG-Lab-BI/Freshwater_Sponges_Sundarban/blob/main/supplemental%20files.pdf). The rarefaction curves plot the diversity indices against the number of sequencing reads, confirming whether the sequencing depth was sufficient to capture most of the microbial diversity across the samples. As indicated by higher asymptotes across all indices, the water samples exhibited greater microbial diversity than the sponge samples. Furthermore, the rarefaction curves of both sample types appeared to reach a plateau at higher sequencing depth, suggesting that the sequencing depth was satisfactory.

**Fig 2:**
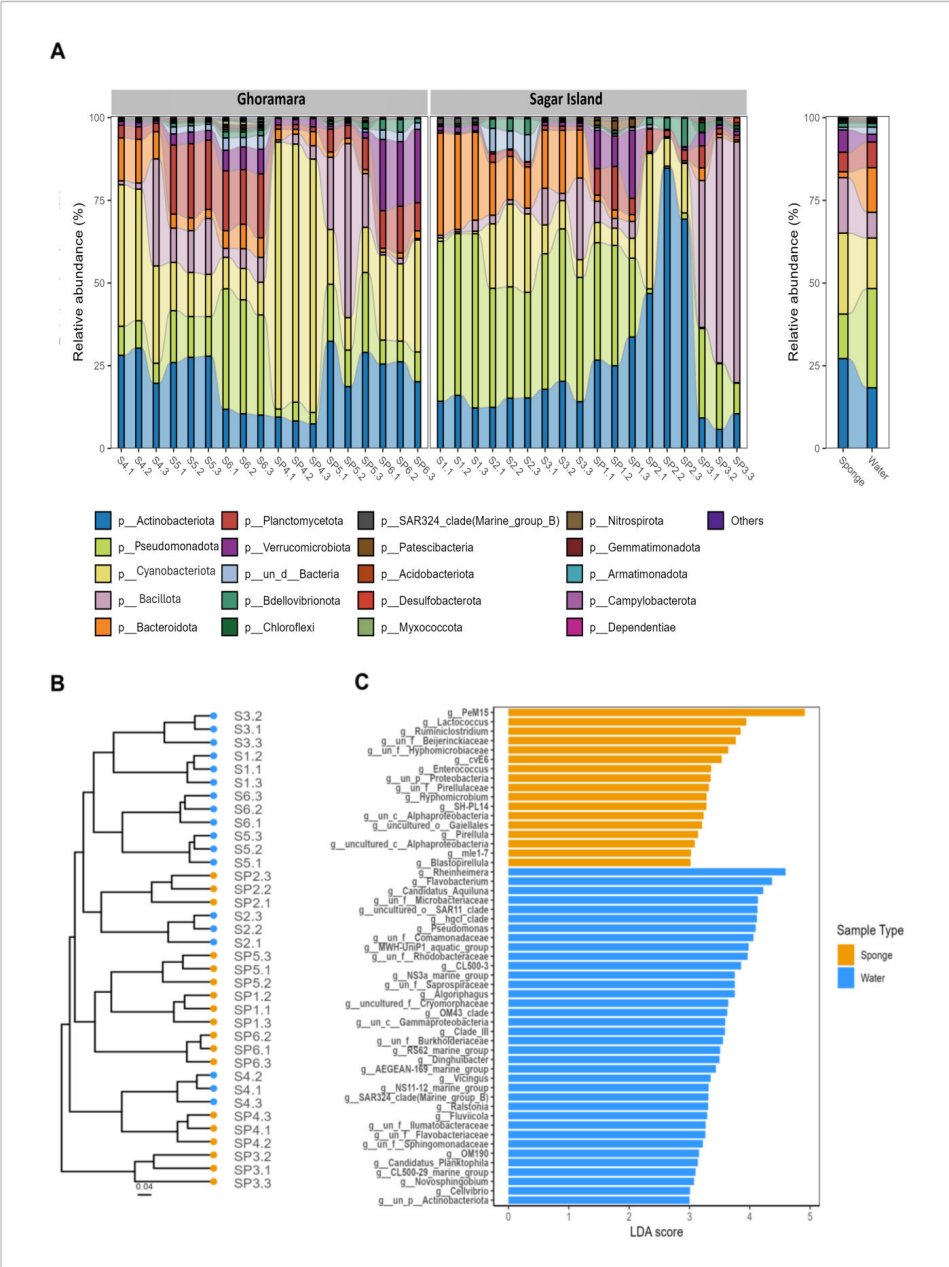
Bacterial diversity across the sponge species and their associated water samples. (**A**) Relative abundance of the top 20 phyla across sampling locations (left) and across sponge and water sample types (right). (**B**) Hierarchical cluster plot based on Bray-Curtis dissimilarity, calculated from Hellinger-transformed abundance data. The dendrogram shows clustering of samples by similarity, with branch lengths reflecting relative dissimilarities among samples. (**C**) The differentially abundant genera with significant differences (*P*-value < 0.05, FDR < 0.05) were identified using Kruskal-Wallis and Wilcoxon tests, followed by linear discriminant analysis (LDA) for effect size estimation. Bars represent LDA scores for each genus, with values indicating the strength of association with sample types.

The stacked bar plot was generated based on the relative abundance of the top 20 phyla across sponge and water samples from Sagar and Ghoramara islands (Fig. 2A). The relative abundance profile of top phyla showed Pseudomonadota (30%) followed by Actinobacteriota (18.3%) and Cyanobacteriota (15.3%) dominating the water samples. In contrast, sponge samples exhibited a greater abundance of Actinobacteriota (27.2%) followed by Cyanobacteriota (24.5%) and Bacillota (16.8%) (see Fig. S6 at https://github.com/AG-Lab-BI/Freshwater_Sponges_Sundarban/blob/main/supplemental%20files.pdf). The OTU abundance-based hierarchical clustering of samples also displayed two distinct clusters corresponding to sponge and water samples (Fig. 2B). Such observations further corroborated the distinction between sponge-associated and free-living microbial communities in water.

The LEfSe analysis recognized several biomarker genera in sponge and water samples, with the Linear discriminant analysis (LDA) scores above the significance threshold set as *P*-value and FDR less than 0.05 (Fig. 2C). The sponge-associated biomarker genera include PeM15 (belonging to actinobacterial family), *Lactococcus*, *Ruminiclostridium*, CvE6 (members of Chlamydiaceae family), *Enterococcus*, *Pirellula,* and *Blastopirellula*. In contrast, *Rheinheimera*, *Flavobactorium*, *Cadidatus_Aquiluna*, *Algoriphagus, Dinghuibacter, Ralstonia, Fluviicola, Pseudomonas, Vibrio, Novosphingobium,* and *Cellvibrio* were differentially abundant in water samples.

**Fig 3:**
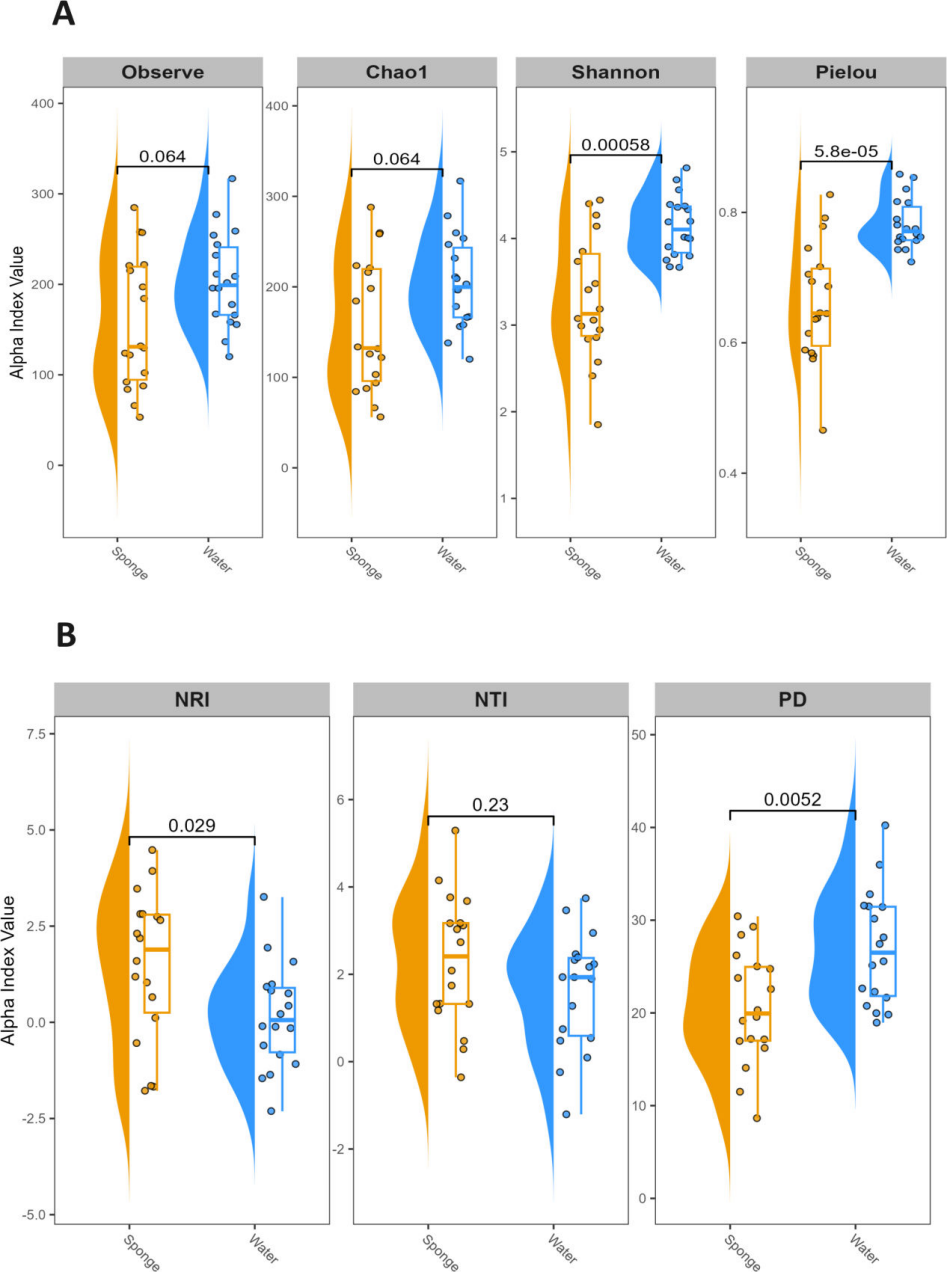
Alpha and phylogenetic diversity of bacterial communities across sample types. (**A**) The alpha diversity indices, comprising observed richness, Chao1, Shannon, and Pielou’s Evenness, depict microbial community richness and evenness across sponge and water sample groups. (**B**) Phylogenetic diversity indices include the Net Relatedness Index (NRI), Nearest Taxon Index (NTI), and Faith’s Phylogenetic Diversity (PD) in groups of sponge and water samples.

The Chao1 and observed indices estimating richness displayed slightly higher values in water samples than in sponges. Likewise, the Shannon and Pielou indices, which estimate the species evenness, were significantly higher (*P* < 0.001) in water samples, indicating more evenly distributed microbial communities (Fig. 3A). The computed phylogenetic indices revealed that sponge samples had greater values of phylogenetic clustering indices, particularly for the Net Relatedness Index (NRI) and Nearest Taxon Index (NTI), compared to water samples (Fig. 3B). However, the Phylogenetic Diversity (PD) is lower in sponge samples than in water samples. The greater NRI and NTI values observed in sponge samples denoted a more phylogenetically clustered taxa found dominantly in sponges. The selective environmental pressures within the host sponge could drive this clustering among the symbionts. The sponges may favor the taxa, which possess specific functional traits crucial for survival within the host’s unique environment.

**Fig 4:**
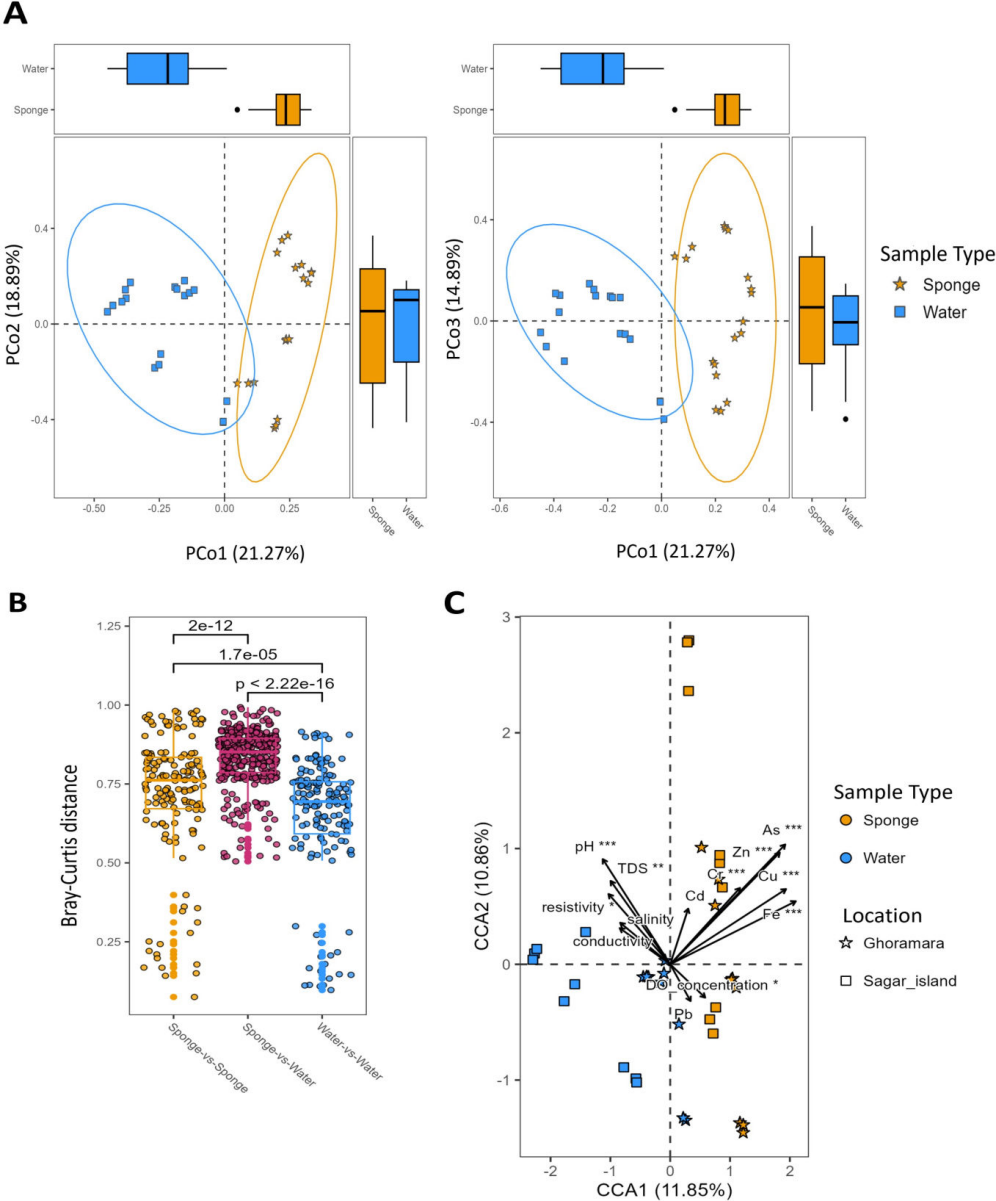
Ordination analyses revealing patterns in community structure across sample types. (**A**) Three-dimensional Principal Coordinate Analysis (PCoA) of bacterial communities using Bray-Curtis dissimilarity across sponge and water samples. (**B**) Distribution of Bray-Curtis distances between and among sample types. (**C**) Canonical Correspondence Analysis (CCA) of bacterial communities with Environmental Variables, including physiochemical parameters and various potentially toxic elements (PTEs) concentrations.

The PCoA plots revealed distinct clustering of sponge and water samples, highlighting significant variations in microbial community composition (Fig. 4A). The first, second, and third principal coordinates explained a substantial proportion of the variance viz. 18.89%, 21.27%, and 14.89%, respectively. The ellipses representing 95% confidence intervals also corroborated the significant differences, underscoring that both sample type and location can influence the composition of the microbial community. Notably, the boxplots beside the axes furnish an impression of the central tendency and dispersion of each sample type, validating that water samples display a relatively greater extent of beta diversity than in comparison to sponge samples.

The Bray-Curtis dissimilarity comparisons in microbial community composition between sponge and water samples showed significant dissimilarities as indicated by the *P*-value substantially less than 2.22*e*^−16^, suggesting distinct microbial community structures (Fig. 4B). Additionally, a comparison using the dissimilarity index within the sponge samples revealed that sponge-associated microbial communities display greater within-group variations than water communities, as indicated by the lower Bray-Curtis dissimilarity index in water samples (Fig. 4B). This suggests a host-specific microbial association in sponges. These findings further suggest distinct variations in microbial diversity influenced by sample type, supporting the idea that microbial communities in sponges and water are compositionally and ecologically distinct.

### Diversity pattern and environmental correlations

The first two ordination axes of the CCA plot (CCA1 and CCA2) collectively accounted for 11.85% and 10.86% of the total variation in the analysis, respectively (Fig. 4C). The environmental variables, denoted by arrows, indicate the directional and quantitative correlations with microbial community composition. Statistically significant (*P* < 0.001) positive correlation between Potentially toxic elements (PTEs), encompassing arsenic (As), zinc (Zn), chromium (Cr), copper (Cu), and iron (Fe), was observed with the sponge-associated microbes. This positive interaction indicates the substantial influence of these PTEs on sponge-associated microbial communities. Conversely, water samples demonstrated a statistically significant negative correlation with the physicochemical parameters, including pH, TDS, resistivity, salinity, conductivity, and DO concentration. Notably, samples from distinct locations such as Sagar Island and Ghoramara showed spatial distinction; however, this separation appeared less pronounced than the distinction between sponge and water samples. These results indicate that sample type has a stronger impact on community composition than geographic location.

The results of the PERMANOVA analysis demonstrated that sponge and water samples accounted for 93.7% of the variation in community composition (*R*² = 0.937, *P* = 0.0001), representing a substantial and statistically significant effect of sample type. The *F*-value reflecting the variation for sample groups was 32.5, further indicating the considerable influence of sample type on shaping the microbial community structure. The residual variation accounted for only 6.3% of the total variability, signifying minimal unexplained differences.

**Fig 5:**
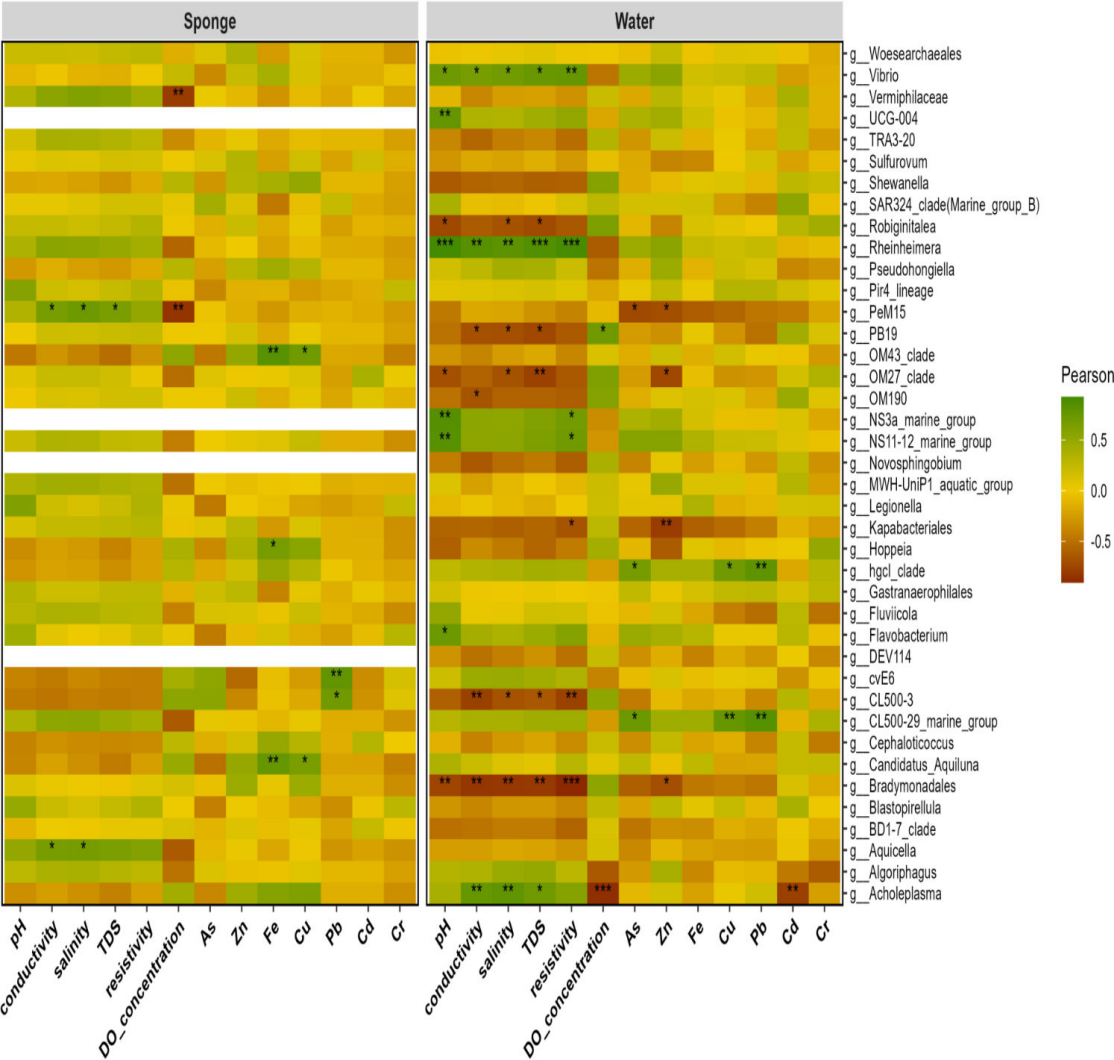
Correlation heatmap of bacterial taxa across sample types and environmental variables. Statistical significance levels are indicated as follows: *P < 0.05; **P < 0.01; ***P < 0.001, ****P < 0.0001.

The correlation study between significantly abundant genera in sponge and water samples with physicochemical parameters and PTE concentrations revealed discrete patterns (Fig. 5). In sponge samples, several enriched genera exhibited positive correlations with PTEs. For example, the members of the OM43 clade (Methylophilaceae family) showed significant positive correlations with iron and copper, *Candidatus_Aquiluna* with both iron and copper, *Hoppeia* with iron, and genera belonging to cvE6 and CLC500-3 with lead. However, only two groups of genera in water samples (hgcl clade and CL500-29 marine group) displayed significant positive correlations with PTE concentrations. Regarding physicochemical parameters, only two sponge-enriched genera, PeM15 and *Aquicella*, showed significant positive correlations with factors such as salinity, conductivity, and resistivity. Nevertheless, the water sample analyses revealed a greater prevalence of genera exhibiting positive correlations with physicochemical parameters, specifically *Rheinheimera*, *Acholeplasma,* and members of Woesearchaeales, which displayed considerable correlations with multiple parameters. These results advocate the strong correlation between sponge-associated microbial communities and PTE concentrations, underscoring sponge-associated microbial composition’s metal tolerance or detoxification capabilities. In contrast, the microbial communities in water samples seem more influenced by physicochemical parameters.

### Functional potential and metabolic insights

**Fig 6:**
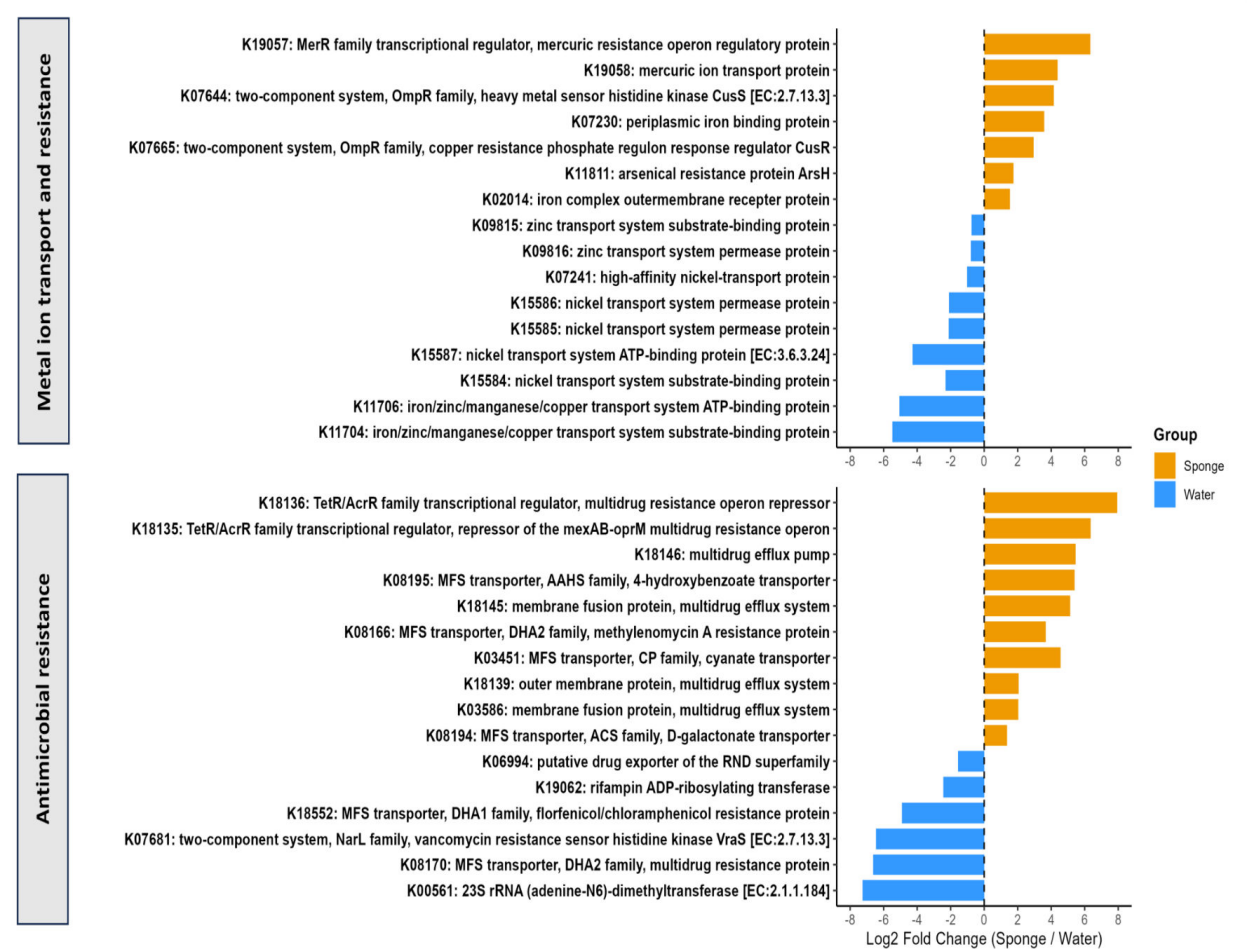
Comparing functional profiles related to metal ion transport and resistance (MITR) and antimicrobial resistance (AMR) classes between water and sponge samples.

The PICRUSt2 followed by ALDEx2 analysis revealed differential abundance of KEGG Orthology (KO) terms associated with metal ion transport and resistance, as well as antimicrobial resistance mechanisms, between bacterial communities in sponge and water samples (Fig. 6). The log2 fold change in abundance for each KO term in sponge vs water samples illustrated an enrichment of genes linked to metal resistance and transport, such as those involved in mercury transport and regulation (K19057 and K19058), arsenic resistance (K11811), and two-component systems of heavy metal sensory kinase and copper resistance regulator (K07644 and K07665) in sponge samples. KO terms with annotation of transport systems for nickel (K07241, K15584, K15585, K15586, K15587) and zinc (K09815, K09816) along with iron/manganese/copper (K11704, K11706) appear as the prevalent in the water samples. This study reflects the adaptive capabilities of sponge-associated microbes with higher metal concentrations, suggesting potential roles for sponges in metal accumulation. In contrast, water samples, including nickel and zinc transport systems, exhibited a greater abundance of certain metal transport genes, specifying a disparate selective pressure in the ambient water. The sponge samples showed a greater abundance of KO terms related to multidrug resistance systems, including efflux systems (e.g., K18145, K18146, K018139, K03586), TetR/AcrR family transcriptional regulators (K18135, K18136), and major facilitator superfamily (MFS) transporters (K08195, K08194) which facilitate bacteria to flush out toxic compounds and resist multiple antibiotics. The water samples exhibit an abundance of MFS transporters (K18552, K08170), vancomycin, florfenicol/chloramphenicol, and rifampin resistance (K07681, K18552, K19062), and 23S rRNA dimethyltransferase (K00561), KO terms. This signifies that sponge-associated microbial communities are more armed to withstand various chemical stresses feasibly due to their specialized partnership with sponges.

## DISCUSSION

The 16S rRNA-targeted metataxonomic data of these sponge species collected from the Sundarbans have not been previously reported. Although diversity analysis of *E. fluviatilis* has been documented before, no next-generation sequencing data for this species is available in the existing records. The bacterial profile of sponges appeared to be distinct from their ambient water. However, the dissimilarity among the sponge species is more significant than within the group difference of ambient water samples (see Fig. S7 to S12 at https://github.com/AG-Lab-BI/Freshwater_Sponges_Sundarban/blob/main/supplemental%20files.pdf). These findings suggest that host sponges selectively associate with bacterial members, and the association largely depends on the sponge species and the host’s habitat.

In our study, relative abundance profiling of top phyla revealed distinct microbial compositions between water and sponge samples, with water samples dominated by Pseudomonadota, Actinobacteriota followed by Cyanobacteriota. In comparison, sponge samples exhibited a higher abundance of Actinobacteriota, Cyanobacteriota, and followed by Bacillota. Gladkikh et al. (19) reported Bacteroidota and Pseudomonadota as the most abundant phyla in two sponge species, *Lubomirskia baicalensis* and *Baikalospongia* sp., endemic to Lake Baikal; similar phyla abundance was observed in lake water. Another study by Itskovich et al. (20) identified that Chlorophyta, Acidobacteria, Bacteroidota, and Actinobacteriota represent the top phyla in both healthy and diseased *Lubomirskia baikalensis* as well as *Baikalospongia intermedia* from Lake Baikal. Moreover, there was an increase in the abundance of phylum Cyanobacteriota in the diseased sponge species. The microbiome study by Belikov et al. revealed that dominant phyla in *L. baicalensis* were reported as Bacteroidota and Pseudomonadota, while the phylum Betaproteobacteria appeared to be associated with diseased sponges. Another study on freshwater sponges *E. carteri* and *C. lapidosa* by Gaikwad et al. (25) revealed significant differences in bacterial communities between these sponge species and water samples. Bacillota, Pseudomonadota, and Cyanobacteriota represented the predominant phyla in *E. carteri,* and Pseudomonadota, Bacteroidota, Actinobacteria, and Cyanobacteriota dominated the water samples associated with this species. In contrast, *C. lapidosa* had Planctomycetota, Cyanobacteriota, and Actinobacteria as abundant phyla. At the same time, the water samples associated with *C. lapidosa* were dominated by Pseudomonadota, Planctomycetota, Cyanobacteriota, and Actinobacteria. Another study employing 16S rRNA targeted as well as shotgun metagenomic studies on freshwater sponge *E. muelleri* showed the abundant phyla associated with the sponge species were Bacteroidota and Pseudomonadota (1).

Although most water samples showed Pseudomonadota as their top abundant phylum, the abundance of phyla varied significantly among the six sponge species. Different phyla-dominated sponge samples collected from Sagar Island: *Spongilla alba* by Pseudomonadota, Actinobacteriota, and Verrucomicrobiota; *Eunapius carteri* by Actinobacteriota, Cyanobacteriota, and Bdellovibrionota; and *Ephydatia fluviatilis* by Bacillota, Pseudomonadota, and Actinobacteriota. Similarly, sponges collected from Ghoramara showed distinct microbial profiles: *Radiospongilla cerebellata* was dominated by Cyanobacteriota, Actinobacteriota, and Pseudomonadota; *Ephydatia meyeni* by Bacillota, Actinobacteriota, and Pseudomonadota; and *Spongilla alba* by Cyanobacteriota, Actinobacteriota, and Verrucomicrobiota. The most abundant genus in this study appeared as Cyanobium, accounting for 23.6% and 14.7% of total bacterial community in sponge and water samples (Fig. S6). Moreover, the water samples exhibited greater bacterial richness and evenness compared to the sponges, while the higher phylogenetic distance values observed in the sponge-associated bacterial community proposed a selective association between the bacteria and the sponge host, with an enrichment of closely related phylogenetic members. The identified biomarker genera associated with sponge and water also supported the distinct differences in the bacterial composition between the two environmental sample types.

Some PTEs such as copper, iron, manganese, nickel, and zinc play essential roles in maintaining cellular structure, regulating gene expression, and supporting antioxidant and neurological functions (70). However, chronic exposure to these elements can disrupt mitochondrial activity, impair enzyme functions, and induce oxidative stress, potentially leading to serious health issues. In contrast, PTEs like arsenic, cadmium, lead, and mercury have no known biological role and are harmful even at low concentrations (71, 72). These PTEs are listed among the World Health Organization’s top 10 chemicals of major public health concern due to their association with neurological damage, kidney dysfunction, cancers, and immune system damage (73). Owing to the heightened susceptibility of children's health to these pollutants and the varying exposure risks through inhalation, ingestion, and dermal contact, it is crucial to frequently screen the PTE levels in water and sediments for sustainable water quality management (74–78). Sessile epifaunas, such as sponges, are primarily responsible for absorbing PTEs in aquatic ecosystems, and the fate of these hazardous elements throughout the benthic biota is influenced by two different mechanisms. Bioaccumulated PTEs either become biologically accessible and biomagnified along the ecological food chain, or they undergo biotransformation or detoxification into latent concretions that attach to lysosomes and are eventually eliminated by the process of excretion (79). Previous studies have shown that sponges can accumulate trace elements by orders of magnitude above levels found in the surrounding waters (40, 48, 80). Notably, several marine sponge species were found to be highly selective in metal sequestration. For example, the marine sponge *Spirastrella cuspidifera* has been shown to store cadmium, chromium, and tin at five to seven times higher than levels found in surrounding seawater (40). The bacterial symbionts of some marine sponges, such as *Entotheonella* sp., are known to sequester metals extracellularly, including arsenic and barium (81), which contribute to metal detoxification. One recent study on the freshwater sponge *Spongilla lacustris* from the sub-arctic Pasvik River in Norway demonstrated promising indication for the ecological relevance of freshwater sponges in polluted aquatic environments (76). The researchers studied considerable accumulation of various pollutants including potentially toxic elements (PTEs) within the sponge tissues, signifying their natural filtering capacity and potential use as bioindicators of environmental contamination. Moreover, the sponge-associated microbial communities were found to be highly diverse and seemed to comprise taxa potentially involved in pollutant degradation, transformation, or sequestration. This suggests that the sponge-microbe consortium may play an active role in the detoxification and biogeochemical cycling of contaminants in freshwater systems. The dual capacity of *S. lacustris* to sequester pollutants and host functionally relevant microbial populations underscores the importance of freshwater sponges not only as passive accumulators of pollutants but also as dynamic participants in health and resilience of the aquatic ecosystem.

Bioaccumulation studies in estuarine, coastal, and marine environments are essential due to their increased exposure to contamination from various metals, chemical pollutants, and organic substances released by urban, industrial, and agricultural activities. Both marine and freshwater sponges have been studied as bio-accumulators of PTEs previously (45, 82–86). Studies on freshwater sponges, including *Ephydatia fluviatilis*, *Ephydatia muelleri*, *Eunapius fragilis*, and *Spongilla lacustris*, have revealed that they can withstand high concentrations of metals such as Ba, Cd, Cr, Mn, Mo, Ni, Pb, and Zn in water (84, 86). These sponges accumulate the metals not in their spicules but in their living tissue and organic skeleton (85). Another study showed sponge *Hymeniacidon perlevis,* which was found to accumulate 44 times the copper, 20 times the zinc, and 16 times the fluoranthene concentrations observed in mussels across the intertidal zones of Lower Normandy coast in France (83). Our study focusing on the freshwater sponges of Sundarban also shows a similar pattern with significantly elevated concentrations of potentially toxic elements (PTEs) found in the sponges compared to the associated water samples. Microbial communities within freshwater sponges are highly diverse and relevant to antimicrobial resistance (AMR). Previous studies demonstrated that horizontal transfer of vancomycin resistance occurs between *Enterococcus faecalis* strains supported by the freshwater sponge *Ephydatia fluviatilis* (87). The microbiome of *Tubella variabilis* was reported to be highly diverse, with the dominant phyla mostly being Pseudomonadota, and it possesses promising antimicrobial activity (26). Likewise, the functional metagenomic study based on Mediterranean high-microbial-abundance sponges identified the sponge-associated taxa *Pseudovibrio*, capable of producing a novel marine-derived β-lactamase that confers ampicillin resistance (88). These results accentuate the relevance of sponge microbiomes as reservoirs of functional antibiotic resistance genes and their capability to affect environmental microbial community structure.

Being filter feeders, the morphology of sponges has adapted to augment water flow efficiency, enabling them to filter out contaminants from their surroundings effectively. The findings of this study further highlight the remarkable adaptability of sponge-associated bacterial communities in response to environmental stressors, particularly in relation to metal ion transport and antimicrobial resistance. The significant enrichment of KEGG Orthology (KO) terms related to metal resistance in sponges advocates a specialized evolutionary strategy that empowers these organisms to withstand in metal-contaminated environments. The occurrence of genes involved in mercury and arsenic transport features the potential of sponges to act as bioindicators of metal pollution, suggesting their role in the bioaccumulation processes. Additionally, the greater abundance of multidrug resistance genes in sponge samples signifies that these bacterial communities are well-armed to thrive under various chemical stresses, possibly due to their close association with the sponges. In contrast, PICRUSt2 analysis revealed that the water samples showed a comparatively higher abundance of specific metal transport genes, suggesting different selective forces in aquatic niches. These outcomes highlight the complex interactions between host sponges and their bacterial partners, which may be influenced by competition with other microbial taxa, nutrient availability, and pollution levels in the ecosystem. Understanding these sponge-microbial dynamics is crucial, as it not only unveils the ecological functions of sponges in coastal and estuarine ecosystems but also hints at implications for conservation strategies and the management of polluted waters. Future research focusing on the functional roles of specific microbial taxa in metal transport and resistance could further elucidate strategies to protect these dynamic and vital ecosystems amidst rising environmental challenges.

### Conclusion

This study offers a clear perspective on the diverse bacterial communities associated with freshwater sponges of the Sundarban delta, as it identifies the specific bacterial taxa associated with each sponge species and their varying ecological roles. The sponges’ ability to accumulate potentially toxic elements (PTEs) and their enriched bacterial communities with metal resistance genes suggest a specialized adaptation to contaminated environments. These findings highlight the potential of sponges and their associated diverse bacterial communities as bioindicators for monitoring metal contamination in estuarine and freshwater ecosystems. Further investigations focusing on the functional roles of these sponge-associated bacterial taxa, explicitly concerning metal detoxification, can provide novel insights into their impacts on ecosystem health and resilience.

## Data Availability

The raw 16S rRNA gene amplicon sequences obtained from this study have been deposited in the Sequence Read Archive of NCBI under a single bioproject with the accession number PRJNA1101956. The Sanger sequencing reads of the D3 domain of the 28S rDNA used for sponge identification have been submitted to the NCBI GenBank database under accession numbers PV413050 to PV413055. The R code, needed to reproduce all analyses can be accessed at https://github.com/AG-Lab-BI/Freshwater_Sponges_Sundarban. Details of sponge and water samples and their physicochemical parameters (Tables S1– and S2), microscopic images of sponge spicule morphology (Fig. S1), alignment of 28S rDNA sequences of sponges (Fig. S2) and phylogenetic tree (Fig. S3) concentrations of potentially toxic elements (Fig. S4), microbial diversity metrics and rarefaction curves (Fig. S5), taxonomic composition at phyla and genus levels in sponge and water samples (Figs. S6– to S12) are available at: https://github.com/AG-Lab-BI/Freshwater_Sponges_Sundarban/blob/main/supplemental%20files.pdf.
